# Conveyance of Sound by the Teeth

**Published:** 1864-04

**Authors:** 


### CONVEYANCE OF SOUND BY THE TEETH.

*To the Editor of the Philadelphia Evening Bulletin*—In the New York *Observer* of Feb. 25, 1864, was an article on the "conveyance of sound," stating, as a fact not generally known, that sound might be transmitted by the TEETH when the ears were closed, almost as distinctly as by the *ears* when they are open.

Wishing to test experimentally this assertion of the *Observer*, I took the end of a long wooden pole (22 feet long) between my the teeth, and requested another person to scratch at the opposite end, when I distinctly heard the sound of the scratching, though when my teeth were removed from contact with the pole it was quite inaudible.

This, in connection with the following incident in Philadelphia a few years ago, suggested to me the injury whether contact with the teeth, as a medium for the communication of sound, might not be resorted to, in some cases with advantage, in the education of the Deaf and Dumb; and in hopes it may lead to some practical benefit to that afflicted class, I send you an account of the incident as follows:

In 1860 I became acquainted with a very sweet deaf and dumb girl, about 15 years old, who was a great favorite with my daughter (of nearly the same age), as indeed she was with all who knew her. One day, my daughter, her deaf and dumb friend, and several other young girls, accompanied by the parents of some of them, visited Fairmount Water Works and while resting in the parlor of the Hotel there, a gentleman with them—the father of one of the girls—called for sherry cobblers for the party, which were furnished, each tumbler being provided with a glass tube by which to draw the liquid into the mouth. When the tumblers were nearly emptied, the air entering with the water into the tubes, produced a gurgling sound. All at once the deaf and dumb girl became greatly exited, laughed vociferously and springing to her feet, and calling by gesture the attention of her companions, pointed, first—into her tumbler, and then to her ears, and then laughed again. As soon as sufficiently composed, she told the other girls, in the manual language of the deaf and dumb, that she heard distinctly, while finishing her cherry cobler, the noise of the water passing through the glass tube from her tumbler to her mouth—the first sound she had ever heard in her life.

When the deafness, in case of deaf and dumb persons, is occasioned by malformation or deficiency in the external structure only of the ear, and the internal parts are complete, it is probable that sound may be communicated successfully by the teeth; but where the internal structure is itself deficient, no means of conveying sound would produce sensation; and it follows that the deaf and dumb person would not hear, whatever means were adopted. Still it would be worth while to try the experiment, an experiment so simple and easy; if it failed the case would be no worse than before; and if it succeeded, the gratification of the patient and friends would be unbounded, while the trouble would be slight. Imagine, for instance, what *exquisite* delight the girl above mentioned might have experienced from the music of a piano or organ communicated, as it might easily have been, by a fixture of metal or wood or any other dense material, attached to any musical intrument, and so formed that she could put one end into her mouth. A common two-foot ruler placed under the lid of a piano would probably convey the sound in this manner distinctly, and a silver bracket screwed to the lid would convey it yet more perfectly.—W.

